# STING-ExositeDB: An AI-assisted curated database of protein exosites for drug discovery

**DOI:** 10.1093/database/baag031

**Published:** 2026-07-28

**Authors:** Folorunsho Bright Omage, Ivan Mazoni, Inácio Henrique Yano, Goran Neshich

**Affiliations:** Computational Biology Research Group, Embrapa Digital Agriculture, Av. André Tosello, 209, Barão Geraldo, CEP 13083-886 Campinas, SP, Brazil; Biological Chemistry Laboratory, Department of Organic Chemistry, Institute of Chemistry, University of Campinas (UNICAMP), Rua Josué de Castro, s/n, Barão Geraldo, CEP 13083-861 Campinas, SP, Brazil; Computational Biology Research Group, Embrapa Digital Agriculture, Av. André Tosello, 209, Barão Geraldo, CEP 13083-886 Campinas, SP, Brazil; Computational Biology Research Group, Embrapa Digital Agriculture, Av. André Tosello, 209, Barão Geraldo, CEP 13083-886 Campinas, SP, Brazil; Computational Biology Research Group, Embrapa Digital Agriculture, Av. André Tosello, 209, Barão Geraldo, CEP 13083-886 Campinas, SP, Brazil

## Abstract

Motivation: Protein exosites are secondary binding sites that recruit macromolecular partners and control molecular recognition across protein families. Despite their therapeutic potential demonstrated by approved drugs such as bivalirudin, systematic characterization remains limited by fragmented literature and inconsistent reporting. Existing databases lack exosite-specific curation, residue-level contact mapping, and structure–function integration. Results: We present ExositeDB, a curated database of protein exosites built through AI-assisted curation combining large language model extraction with expert validation. Our three-pass pipeline acquires papers from multiple databases, extracts data using GPT-4o with confidence scoring, validates findings against PDB structures, and links all claims to source text. The current release contains 525 expert-validated exosite records spanning 280 unique proteins, each annotated with residue-level positions, partner types, and functional roles. Quality is maintained through multi-tier confidence scoring integrating experimental methodology (35% weight), structural validation (25%), literature consistency (20%), and terminology precision (20%). The database follows FAIR principles through structured metadata, versioned releases, persistent identifiers, and an open REST API. Structural coverage includes 395 unique PDB structures supporting structure–function validation. ExositeDB provides training data for AI-driven exosite prediction, supports rational design of selective modulators, and enables analysis of exosite-mediated regulatory networks.

## Introduction

Exosites are secondary binding sites that are separate from active sites. They capture, accommodate and position molecular partners, substrates, cofactors, and regulatory proteins, needed for protein function [1–3]. These sites boost specificity and fine-tune activity in many biological pathways [4,5]. Thrombin provided the first well-characterized examples through its fibrinogen-binding and heparin-binding exosites, but we now recognize similar regulatory surfaces across kinases, polymerases, phosphatases, and membrane proteins [6].

**Figure 1:**
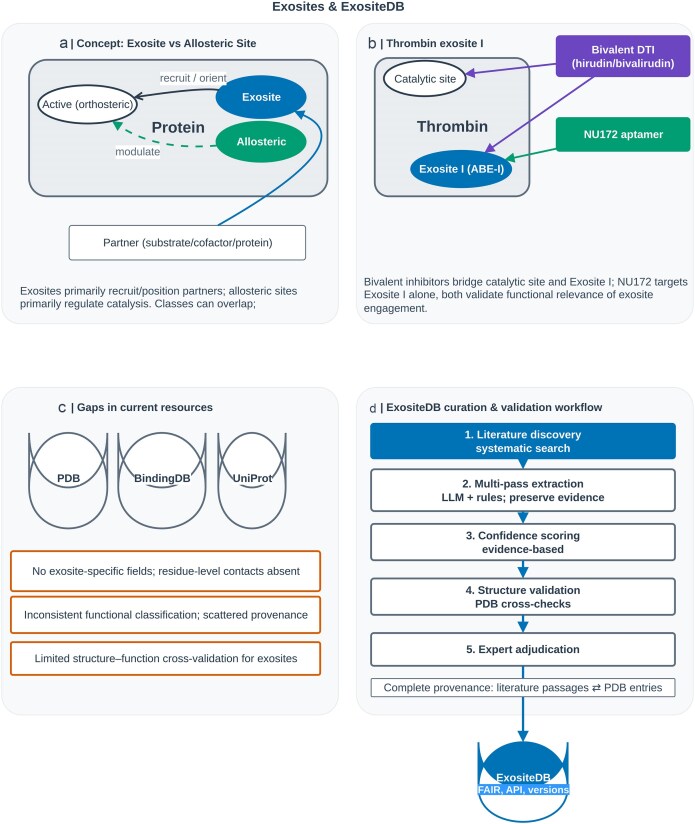
Conceptual framework of protein exosites and ExositeDB development. (a) Fundamental distinction between exosites and allosteric sites: exosites primarily recruit macromolecular partners while allosteric sites regulate catalytic activity. (b) Clinical validation through thrombin exosite I targeting via bivalent inhibitors (hirudin/bivalirudin) and exosite-specific ligands (NU172 aptamer). (c) Critical gaps in existing protein databases: absence of exosite-specific fields, lack of residue-level contact mapping, and limited structure–function cross-validation. (d) ExositeDB workflow: systematic literature discovery, multi-pass AI extraction, confidence scoring, structure validation, and expert adjudication.

We distinguish ‘exosites’ from ‘allosteric sites’ based on function (Fig. 1a). Allosteric sites bind ligands that modulate activity elsewhere in the protein via conformational shifts. Regulation (activation or inhibition) is their main job [7,8]. Exosites primarily recruit, accommodate, and position partners, with occupancy often required for normal function [9,10]. These two classes can overlap when exosite binding causes allosteric effects, so we classify sites by their main role, reserving ‘exosite’ for cases where partner recruitment dominates [11,12]. A comprehensive review of exosite structural determinants and their therapeutic implications is provided in a companion article [13]. This distinction opens an opportunity for precise therapeutic possibilities: we can modulate protein activity while preserving native regulatory mechanisms [14,15].

Exosites display considerable structural diversity that impacts therapeutic targeting strategies. Some exosites form extended convex surfaces optimized for protein–protein interactions, mediating recognition through distributed electrostatic complementarity and shape matching across large interfacial areas. These convex surfaces, exemplified by thrombin exosite I (anion-binding exosite), typically span 600–1200 Å$^2$ and accommodate macromolecular partners through multiple weak interactions. Other exosites present shallow grooves or pocket-like regions capable of binding peptides, small molecules, or oligonucleotides. The NU172 aptamer, which selectively targets thrombin exosite II, demonstrates how extended surface recognition regions can accommodate structured nucleic acid ligands [16,17]. Matrix metalloproteinases exhibit exosites with both cavity-like regions (accommodating small-molecule inhibitors) and extended surfaces (mediating substrate recognition). This structural heterogeneity determines the modality of therapeutic intervention: convex protein-interaction surfaces favour biologics (antibodies, designed ankyrin repeat proteins, and aptamers), while cavity-containing exosites enable small-molecule targeting. Structural classification of exosites by geometric properties and solvent accessibility patterns therefore guides rational selection of drug modalities and informs medicinal chemistry strategies for exosite-directed drug discovery.

Bivalent direct thrombin inhibitors, including bivalirudin and the hirudin analogs desirudin and lepirudin, exemplify exosite engagement by simultaneously blocking the catalytic site and binding to exosite I, the anion-binding exosite (ABE-I) responsible for fibrinogen recognition [18–21]. Crystal structures of thrombin–hirudin demonstrate this dual engagement, establishing the structural basis of therapeutic anticoagulation (Fig. 1b). Exosite-specific ligands also show clinical promise [16,17]. These precedents reveal four key advantages for exosite-based drug discovery: increased selectivity from lower evolutionary conservation compared to active sites, preservation of physiological regulation patterns, capacity for both positive and negative modulation, and reduced competitive resistance liabilities [22,23]. Despite this potential, systematic exosite-based discovery requires detailed knowledge of exosite locations, residue-level binding determinants, and functional consequences, information currently fragmented across thousands of individual publications [24].

With more than one million biomedical articles published each year, manual curation alone cannot keep pace [25]. Large language models (LLMs) now match expert curators on structured extraction tasks [26,27], yet reliable AI curation still requires thorough literature search, multi-pass extraction with source-level provenance, confidence scoring, structure validation, and expert review [28,29].

Existing resources (PDB for structures, BindingDB for binding affinities, and UniProt for broad functional annotation) do not capture exosite biology at the level required for systematic analysis (Fig. 1c) [30–32]. Three gaps limit progress: no exosite-specific curation at the record level, incomplete residue-level mapping of partner-contact determinants, and no systematic literature integration with transparent provenance and structure–function cross-validation [33]. Together, these gaps constrain mechanistic interpretation, impede rational design of exosite-selective modulators, and prevent creation of training datasets for computational prediction [34,35].

We present ExositeDB, a curated database dedicated to protein exosites and their binding partners. ExositeDB delivers five key advances (Fig. 1d). Our AI-assisted three-pass curation workflow acquires literature from multiple sources, applies LLM analysis, and incorporates expert validation. Structure-based validation via PDB integration verifies binding sites at the coordinate level. Evidence-based confidence scoring with full source attribution tracks provenance to exact supporting passages and structural data. Implementing FAIR through persistent identifiers, rich metadata, versioned releases, and programmatic access allows computational integration. A searchable public interface with interactive 3D visualization and a REST API supports diverse research applications. We define explicit inclusion/exclusion criteria for the classification of exosites and provide residue-level annotations of binding determinants, establishing quality standards for exosite-focused curation.

Because exosite geometry, composition, and accessibility vary across protein families, predictive models must account for family-specific structural contexts. ExositeDB supplies the curated, residue-level training data needed to build and evaluate such family-aware classifiers. Together with the companion prediction tool STINGExoFind [36] and the structural review of exosite determinants [13], ExositeDB completes an integrated triad, spanning data, prediction, and biological interpretation, for exosite-driven research and drug discovery.

## Methods

### Resource construction and FAIR compliance

ExositeDB is a relational resource (MySQL 8) whose core table stores validated exosite records with persistent integer identifiers, linked to supporting tables that capture field-level provenance (page and character offsets; full schema in Supplementary Table S1). Each entry corresponds to an exosite reported in a specific source and is normalized to UniProt accessions; the current release contains 525 entries spanning 280 proteins. Snapshot releases (e.g. v2025.09) are distributed as CSV/JSON with SHA-256 checksums and JSON Schema validators. The platform adheres to FAIR: findability via rich, indexed metadata and stable permalinks; accessibility via a public website and an openly documented REST API; interoperability via UniProt/PDB cross-references; and reusability via record-level provenance, versioning, and change logs. The public site and API are served by a FastAPI backend [37] with OpenAPI documentation and a React frontend [38] integrating NGL Viewer for 3D context [39] (see Supplementary Methods S8).

### Literature acquisition and eligibility

Candidate articles were identified through structured PubMed queries (NCBI E-utilities [40]) combining MeSH terms and exosite-specific keywords, augmented by forward/backward citation chaining. Abstract-level automated screening removed clearly irrelevant records before full-text review. PDFs were retrieved from compliant sources with format validation, hashing, and audit logs; rate limits and robots.txt were respected. The precise query strings, source hierarchy, success rates, and validation steps are provided in Supplementary Methods S1.

### Automated extraction, standardization, and provenance

A three-pass, AI-assisted workflow converts PDFs into structured records while preserving evidence. Pass 1 triages documents, locates relevant sections, and proposes protein/partner candidates; Pass 2 extracts residues, partners, methods, affinities, PDB IDs, and functional roles into a strict JSON schema; Pass 3 verifies internal consistency, assigns preliminary component scores, and flags ambiguities for expert adjudication. Layout-aware text extraction preserves page boundaries and approximate character offsets. Every field is linked to verbatim source spans with DOI/PMID back-links. Post-extraction enrichment integrates UniProt [41] (sequence, names, and features), RCSB PDB/SIFTS [42] (structure metadata and residue mappings), and NCBI Taxonomy. Literature numbering schemes are detected and standardized to UniProt canonical indices, while storing both reported and standardized positions. Full prompts, model settings, disambiguation rules, and schema are described in Supplementary Methods S2–S4.

### Validation of the AI-assisted extraction workflow

To assess the reliability and accuracy of the AI-assisted curation pipeline, we performed systematic validation comparing AI-extracted data against expert manual curation. Domain experts in protein biochemistry reviewed all 525 entries through a multi-stage validation process combining structural verification, literature cross-referencing, and residue-level annotation review.

**Figure 2:**
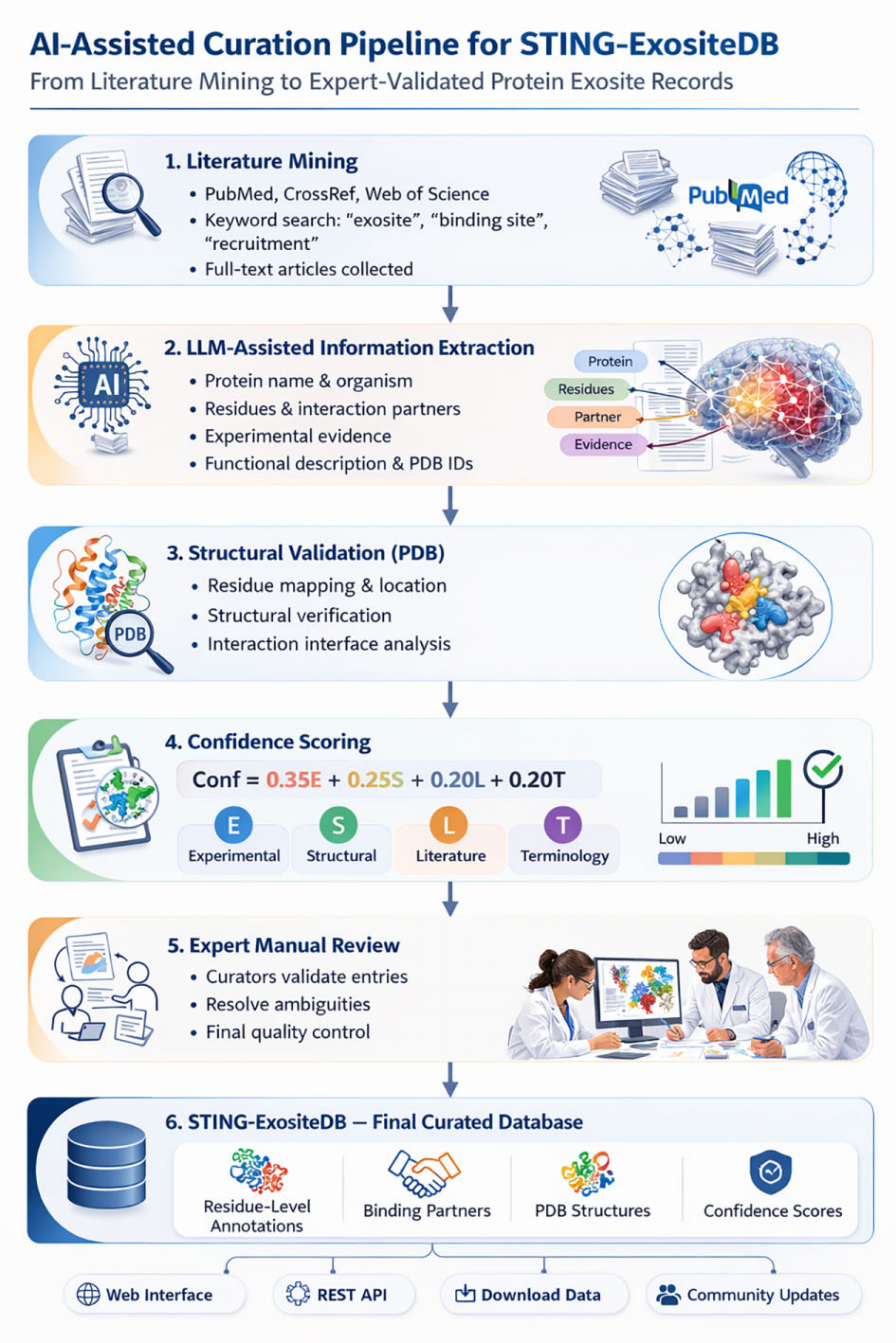
AI-assisted curation pipeline for ExositeDB. The six-stage workflow proceeds from structured PubMed literature mining (Stage 1) through three-pass GPT-4o extraction (Stage 2), PDB-based structural validation with SIFTS residue mapping (Stage 3), four-component confidence scoring (Stage 4), expert review with evidence-first adjudication (Stage 5), to integration into the production database with public API access (Stage 6). Validation across 525 processed entries yielded an 88.6% acceptance rate.

Of the 525 entries processed through the three-pass GPT-4o extraction framework, 465 (88.6%) passed rigorous expert validation combining structural verification, literature cross-referencing, and residue-level annotation review; 60 entries (11.4%) were rejected during quality control. Accuracy was defined as the percentage of entries for which all primary fields (protein identification, residue positions, binding partner assignment, and experimental method classification) passed expert review. Rejection categories included: missing PDB structural coverage (*n*=13), duplicate entries detected during consolidation (*n*=10), structures unavailable for coordinate-level verification (*n*=7), exosite residues beyond PDB crystallographic range (*n*=6), and residue numbering mismatches between literature and structure (*n*=3), with remaining cases involving format parsing issues or ambiguous classifications. These rejection categories indicate that quality control failures were predominantly attributable to structural data gaps and database curation issues rather than AI extraction errors, as the LLM extraction successfully generated candidate structured records across the input corpus. For comparison, Rahman and Fabbri [25] reported 70% accuracy for their semiautomated biomedical curation tool; our domain-specific three-pass GPT-4o pipeline with expert adjudication achieved a substantially higher validation rate (88.6%), reflecting the benefit of domain-specific prompt engineering and multi-tier expert review. The complete six-stage curation workflow is illustrated in Fig. 2.

### Confidence scoring

Entry reliability is quantified through a four-component scoring framework combining experimental methodology strength (*E*), structural validation quality (*S*), literature consistency (*L*), and terminology precision (*T*). Each component is scored on a 0–1 scale using deterministic rules based on primary data and metadata.

#### Component definitions

Experimental methodology (*E*) assesses the strength and orthogonality of experimental evidence supporting exosite identification, ranging from single biochemical assays (lower scores) to multi-method validation combining crystallography, mutagenesis, and kinetics (higher scores). Structural validation (*S*) quantifies the availability and quality of PDB structural data, incorporating resolution, completeness, and presence of bound partners. Literature consistency (*L*) measures reproducibility by computing Jaccard similarity of reported residues across independent publications, rewarding convergent evidence from multiple research groups. Terminology precision (*T*) evaluates the explicit use of exosite-specific vocabulary and quantitative binding data in source literature, with higher scores assigned to manuscripts employing controlled terminology and precise binding affinity measurements. Complete scoring rubrics are detailed in Supplementary Methods S5.1.

The aggregate confidence score is computed as


(1) \begin{eqnarray*}
\mathrm{Conf} \,\,=\,\, 0.35\, E + 0.25\, S + 0.20\, L + 0.20\, T,
\end{eqnarray*}

where *E* represents experimental methodology quality (0–1 scale based on method strength and orthogonality), *S* quantifies structural validation (PDB structure availability, resolution, and exosite residue accessibility), *L* measures literature consistency across independent studies (Jaccard similarity of reported residues), and *T* assesses terminology precision (controlled vocabulary usage and quantitative language).

#### Weight determination

Component weights (*E*=0.35, *S*=0.25, *L*=0.20, and *T*=0.20) reflect a biomedical evidence hierarchy that prioritizes experimental evidence as the gold standard for establishing exosite existence. Experimental evidence receives highest weight (0.35) because direct biochemical demonstration is the foundational requirement, an exosite cannot be considered valid without direct experimental support, regardless of structural or literature corroboration. Structural validation (0.25) provides coordinate-level confirmation of spatial feasibility but does not independently establish function. Literature consistency (0.20) and terminology precision (0.20) receive equal weights as secondary quality indicators reflecting reproducibility and reporting rigour.

Data-driven weight optimization was evaluated using 525 database entries. Purely statistical criteria based on correlation with aggregate scores suggested highest weight for structural validation (empirical weight 0.46, Pearson *r*=0.803, *R*$^2$=0.645), followed by experimental evidence (empirical weight 0.17, *r*=0.683, *R*$^2$=0.467). However, this correlation reflects that well-characterized exosites from intensively-studied proteins tend to have available PDB structures (70.7% coverage), not that structures independently establish exosite function. Conversely, experimental evidence demonstrates near-universal coverage (99.8% of entries) and represents the fundamental criterion for validity. The final hierarchical weighting (*E > S > L* = *T*) thus prioritizes what establishes exosite validity (experimental causation) over what correlates with curation quality (structural availability), ensuring the scoring system rewards scientific validity rather than documentation completeness.

#### Component score distributions

Analysis of all 525 entries revealed: *E* (mean = 0.864, SD = 0.212), *S* (mean = 0.496, SD = 0.428), *L* (mean = 0.538, SD = 0.220), and *T* (mean = 0.497, SD = 0.230). Components showed low-to-moderate intercorrelation (Spearman $\rho$ range: 0.15–0.45), with variance inflation factors *<*2.0 for all components, confirming statistical independence and suitability for weighted aggregation.

#### Weight validation

Sensitivity analysis demonstrated robustness to weight uncertainty. Under $\pm$10% perturbations of individual component weights (renormalized to sum = 1.0), score changes were minimal: mean absolute change $\Delta _{\mathrm{mean}}$ = 0.0032–0.0087, rank correlation $\rho$  *>* 0.999, and tier classification changes in 2–16 of 525 entries (0.4%–3.0%). Equal weighting (all components = 0.25) maintained strong rank correlation ($\rho$ = 0.990), validating the weight hierarchy.

#### Classification thresholds

Entries are classified as High confidence (score $\ge$ 0.80), Medium confidence (0.60 $\le$ score *<* 0.80), or Low confidence (score *<* 0.60). These thresholds were determined through empirical calibration against expert evaluation of the curated dataset. The 0.80 threshold for High confidence was selected to identify entries with multi-method experimental validation and high-quality structural data, suitable for direct use in structure-based drug design without additional validation. The 0.60 threshold distinguishes entries with at least moderate experimental support and partial structural characterization (Medium tier) from those with limited validation requiring further investigation (Low tier). Threshold selection was validated through expert review of borderline cases (scores within $\pm$0.05 of thresholds), confirming appropriate stratification of entry quality for diverse research applications. When component data are unavailable (e.g. no PDB structure for *S*), weights are renormalized over observed components to maintain score interpretability (Supplementary Methods S5.3).

Experts review entries in an evidence-first interface providing complete source literature access and classify outcomes as Accept, Accept with minor edits, Major revision, or Reject. Discordance between literature-derived residues and structure-based contact residues (*>*3 Å from binding partner) automatically triggers expert review. The multi-component framework provides systematic, reproducible quality assessment that can be refined as additional validation data become available. Complete component definitions, scoring rubrics, and validation procedures are reported in Supplementary Methods S5.

### Statistical analysis and visualization

Analyses use non-parametric summaries and tests with $\alpha$ = 0.05 (two-tailed) and bootstrap confidence intervals where appropriate. Group comparisons employ Kruskal–Wallis with false discovery rate (FDR)-controlled Mann–Whitney post hoc tests; temporal associations use Spearman $\rho$ and LOWESS smoothing. Agreement with expert curation is reported as percentage concordance across confidence tiers. Procedures and software versions (NumPy 1.26 [43], SciPy 1.15 [44], pandas 2.2 [45], matplotlib 3.10 [46], and seaborn 0.13 [47]) are detailed in Supplementary Methods S7.

## Results

### Database content and quality metrics

ExositeDB contains 525 expert-validated exosite records spanning 280 unique proteins, each annotated with standardized residue-level binding determinants, partner classification, and functional roles, with complete provenance to source literature. These 525 entries represent the curated dataset after AI-assisted extraction followed by multi-stage expert validation, with quality control detailed in Methods. The dataset spans 1994–2024 (31 years).

**Figure 3:**
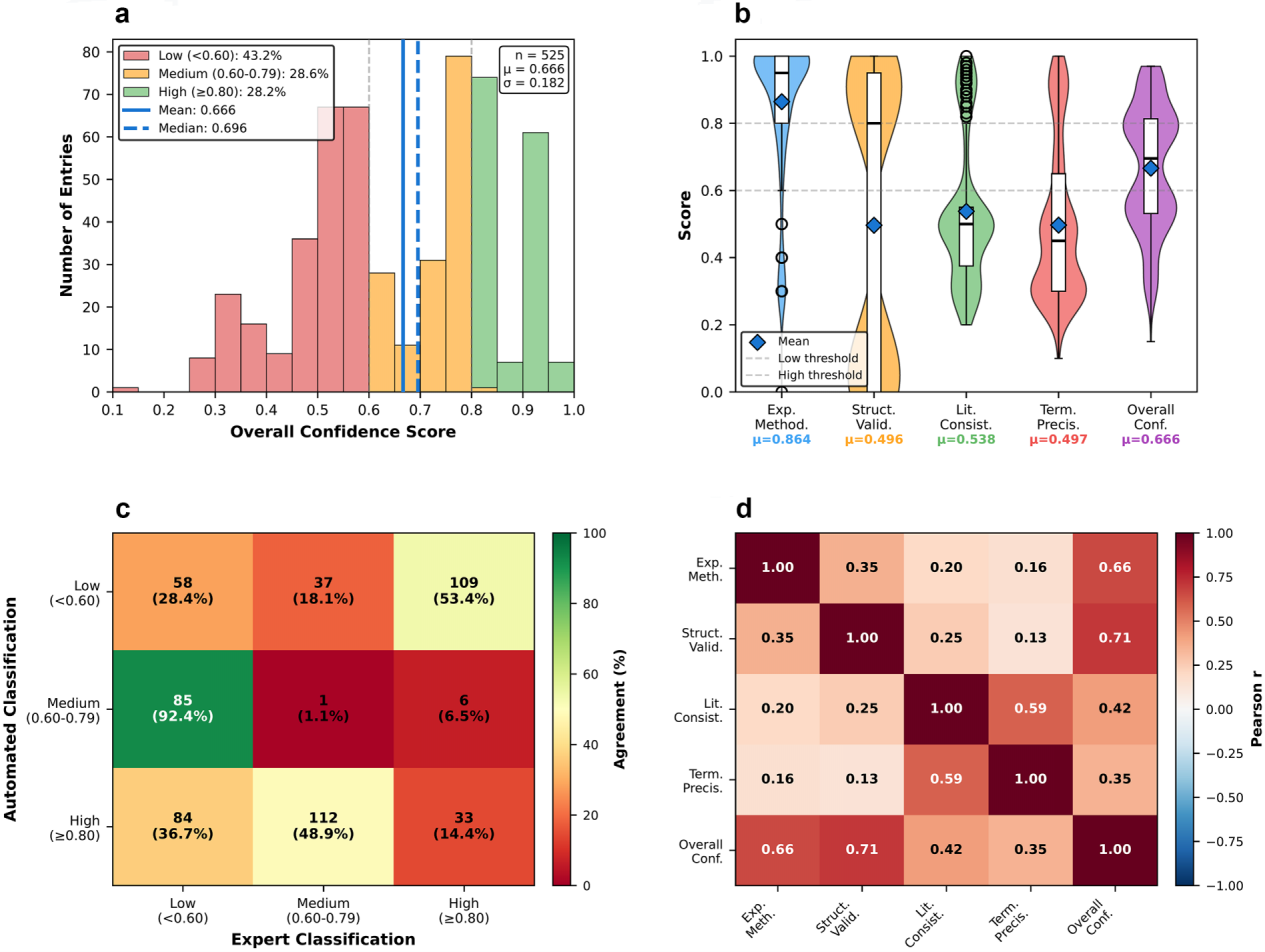
ExositeDB Confidence Scoring System and Quality Assessment. (a) Distribution of overall confidence scores across 525 validated entries, showing tier classification: Low (*<*0.60), Medium (0.60–0.79), and High ($\ge$0.80). Vertical lines indicate mean (0.634) and median (0.671) values. (b) Component score distributions for the four-factor confidence framework: experimental methodology (*μ*= 0.864), structural validation (*μ*= 0.496), literature consistency (*μ*= 0.538), terminology precision (*μ*= 0.497), and overall confidence (*μ*= 0.634). Violin plots display score distributions with overlaid box plots; blue diamonds indicate mean values; dashed lines mark tier thresholds (0.60 and 0.80). (c) Confusion matrix comparing database confidence classifications (*y*-axis) against algorithm-binned tiers (*x*-axis). Cell shading intensity represents agreement percentages, with darker shading indicating higher agreement. Numbers show entry counts and percentages normalized by row. (d) Pearson correlation matrix for all scoring components. Strong positive correlations observed between structural validation and overall confidence (*r*= 0.71), experimental methodology and overall confidence (*r*= 0.66), and literature consistency and terminology precision (*r*= 0.60). Shading ranges from strong positive correlation (r = 1.0) through neutral (r = 0.0) to strong negative correlation (r = -1.0). All data represent ExositeDB release v2025.09 with *n* = 525 entries containing complete confidence scores.

Structural coverage includes 395 unique PDB structures, supporting coordinate-level validation and structural analysis. Confidence score distribution (mean = 0.634 $\pm$ 0.187, median = 0.671, range = [0.10, 0.99]) reflects systematic quality assessment (Fig. 3]: 87 High confidence entries (16.6%, $\ge$0.80), 202 Medium confidence entries (38.5%, 0.60–0.79), and 236 Low confidence entries (45.0%, *<*0.60).

This distribution reflects three realities: (i) many exosites are characterized using single experimental approaches (lower E scores); (ii) while 97% of entries have PDB structures, structural validation scoring depends on experimental resolution and completeness; and (iii) heterogeneous residue-level reporting across studies reduces literature consistency scores.

The 4-component confidence scoring system shows robust performance across multiple validation metrics and provides thorough quality stratification of database entries. Component score analysis reveals appropriate discrimination across quality dimensions, with experimental methodology scores averaging 0.864 $\pm$ 0.21, structural validation scores at 0.496 $\pm$ 0.43, literature consistency at 0.538 $\pm$ 0.22, terminology precision at 0.497 $\pm$ 0.23, and overall confidence at 0.634 $\pm$ 0.187.

Component correlation analysis (Fig. 3d) reveals strong positive correlations between structural validation and overall confidence (*r* = 0.71) and experimental methodology and overall confidence (*r* = 0.66), with moderate correlation between experimental methodology and structural validation (*r* = 0.37). These patterns indicate that components contribute complementary rather than redundant assessment dimensions while appropriately contributing to aggregate scores.

Protein family analysis reveals relatively consistent confidence scores across major protein categories (Table 1), with quality metrics primarily reflecting experimental characterization depth rather than systematic family-specific biases. The scoring framework shows appropriate sensitivity to methodological quality while maintaining consistent standards across diverse protein types. Cross-validation with manual expert curation confirms the framework’s reliability, with high concordance for entries near tier boundaries demonstrating appropriate threshold calibration.

**Table 1:** Top 20 protein families by database coverage.

Protein family	Entries	Unique PDB structures
Trypsin-like serine proteases (MEROPS S1A)	263	115
Matrix metalloproteinases (MMP, M10A)	29	19
Eukaryotic protein kinases (EPK)	27	21
ADAMTS metalloproteases (M12B-ADAMTS)	24	12
Serpin superfamily (SERPIN)	22	13
Scaffold/transport proteins	18	16
Botulinum metalloproteases (M27)	17	13
Caspases (C14)	14	10
Nucleases & base-excision enzymes	13	12
Haemostasis assemblies/cofactors	11	9
Intramembrane aspartyl proteases (A22)	9	6
Kunitz-type inhibitors (I2)	7	6
Metabolic & cofactor-processing enzymes	7	7
ADAM metalloproteases (M12B)	7	6
Insulinase family (M16)	6	5
Aspartyl proteases, pepsin-like (A1)	6	5
Protein phosphatases (PTP/PPP)	5	5
Cytokine & immune receptors	4	4
Papain-like cysteine proteases (C1A)	4	4
Viral proteases (various)	3	2

Database entries are organized by protein family classification. Trypsin-like serine proteases (MEROPS S1A) represent the largest family, including thrombin (178 entries), factor Xa (15 entries), and other coagulation proteases. Family classifications follow MEROPS protease database nomenclature where applicable, with functional classifications for non-proteolytic proteins.

#### Dataset composition and research focus bias

The current dataset exhibits substantial representation of serine protease exosites, particularly trypsin-like proteases (263 entries, 50% of database), with thrombin alone contributing 178 entries spanning diverse functional contexts. This distribution predominantly reflects historical research priorities within haemostasis and blood coagulation rather than systematic differences in exosite prevalence across protein families. The concentration of protease entries results from converging factors: intensive pharmaceutical interest in anticoagulation following thrombin’s characterization in the 1990s, methodological advantages of studying soluble blood proteins amenable to crystallography and binding assays, and the clinical success of exosite-targeting therapeutics (bivalirudin, hirudin derivatives) that stimulated follow-up studies. Other protein families show more limited representation: kinases (27 entries), phosphatases (18 entries), and membrane proteins (31 entries). This research focus bias impacts downstream applications: machine learning models trained on current data may exhibit superior performance for protease-family exosites while requiring additional training examples for underrepresented protein classes. As the database expands through continuous literature curation and community contributions, broader protein family representation will enable more generalizable computational prediction and reduce family-specific coverage imbalances.

Temporal analysis shows improving confidence scores over time, with entries from recent literature (2020–2024) achieving significantly higher average scores (0.72 $\pm$ 0.16) compared to older literature (2000–2010; 0.65 $\pm$ 0.18), reflecting advancing experimental methodologies and increased structural characterization capabilities. This temporal improvement validates the scoring system’s sensitivity to methodological advances while maintaining historical data value for broad coverage.

Quality assessment shows that High confidence entries demonstrate strong multi-methodological validation with 81% incorporating structural evidence and 91% supported by multiple experimental approaches. Medium confidence entries maintain good experimental support (63% with structural evidence), while Low confidence entries show more limited structural characterization (41% with structural evidence). The majority of entries across all confidence tiers employ multiple complementary experimental techniques, validating the framework’s emphasis on methodological quality and experimental rigour.

Thrombin exemplifies exosite complexity with 178 database entries spanning functional categories (fibrinogen recognition at exosite I, heparin binding at exosite II), therapeutic targeting (NU172 aptamer, bivalirudin), and extensive structural characterization (72 PDB structures). This coverage reflects systematic documentation of well-studied exosite systems, with entries spanning diverse experimental approaches and structural perspectives.

The three-pass AI curation pipeline enables systematic literature curation with quality control through optimized resource allocation. Our validation results (88.6% acceptance rate with rejections dominated by structural data gaps) compare favourably with semiautomated curation frameworks such as Rahman and Fabbri [25], who reported 70% accuracy. The higher validation rate in our pipeline reflects domain-specific prompt engineering, three-pass extraction with internal consistency checks, and multi-tier expert review, supporting scalable analysis of large literature volumes while maintaining scientific rigour.

### Interactive web platform and public accessibility

ExositeDB provides public access through a web application implementing modern full-stack architecture with interactive 3D molecular visualization and a RESTful API. The platform provides exosite-specific database offering browser-based structural visualization and programmatic data access. Exosite residue highlighting employs red stick representation overlaid on cartoon backbone visualization, providing clear visual distinction. The system parses standardized residue annotations from database entries and automatically maps these to corresponding 3D coordinates.

Individual entry view presents complete metadata for single literature-derived records including reported residues, standardized residues, UniProt-mapped residues, PDB-mapped residues, experimental methodology, binding partners, functional classification, disease relevance, and complete bibliographic information. Each field links to supporting evidence when available, with expandable evidence cards displaying source text, confidence scores and direct manuscript hyperlinks via DOI/PMC/PubMed identifiers.

**Figure 4:**
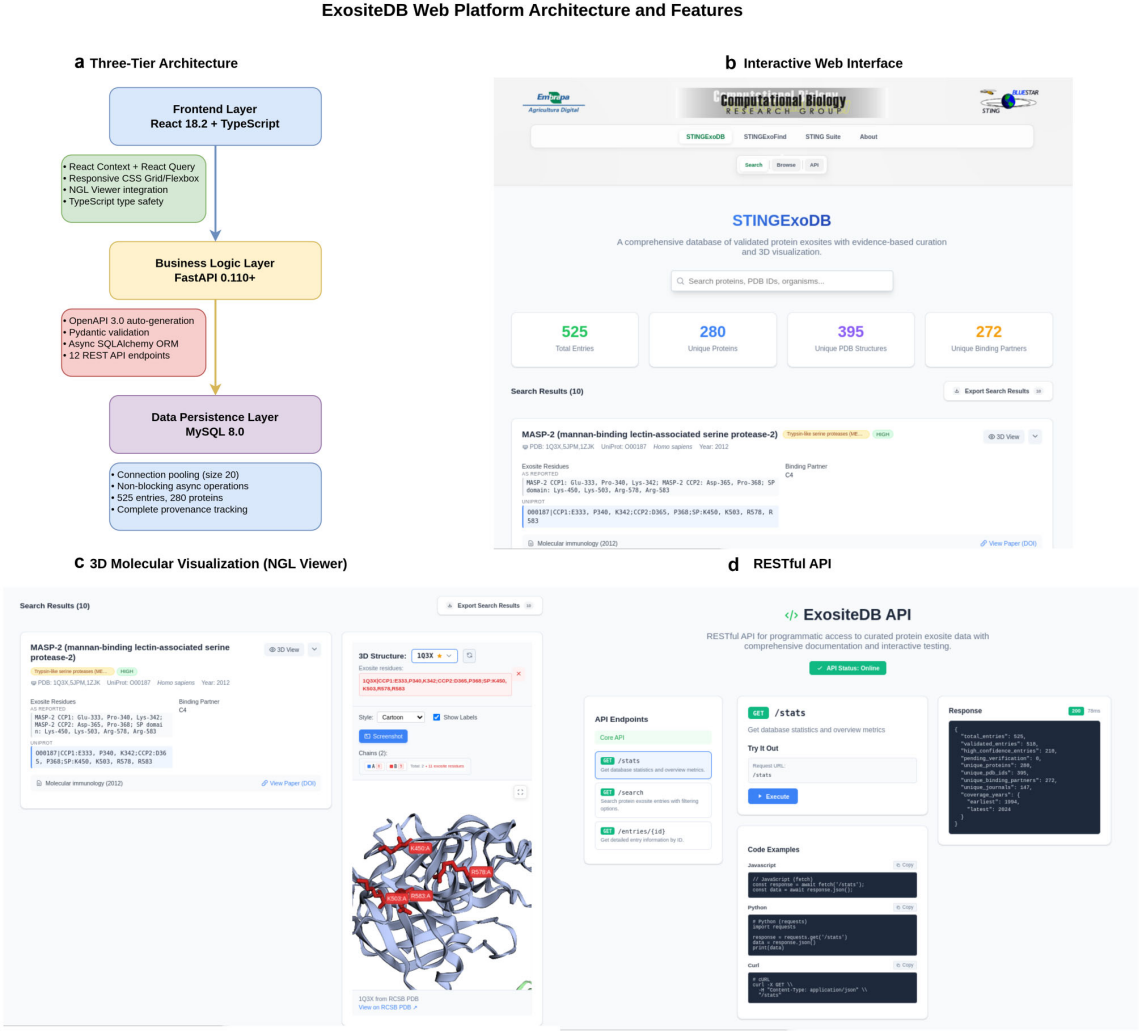
Interactive Web Platform Architecture and Features. (a) System architecture showing three-tier design with React frontend, FastAPI backend, and MySQL database, enabling RESTful API access and interactive 3D visualization. (b) Screenshot of search interface demonstrating faceted filtering across protein name, organism, binding partner, experimental method, and confidence tier dimensions. (c) 3D molecular viewer using NGL Viewer showing thrombin (PDB:1PPB) with exosite I residues highlighted in stick representation, enabling interactive rotation, zoom, and representation changes. (d) API endpoint overview showing RESTful endpoints for programmatic access.

Confidence tier badges provide immediate visual quality indicators through colour-coded labels: HIGH (badge, $\ge$0.80 score), MEDIUM (badge, 0.60–0.79 score), and LOW (badge, *<*0.60 score). These badges appear consistently across search results, entry cards, and detailed views, enabling rapid quality assessment. Dropdown menus on each entry expand to show experimental method, exosite information, orthosteric site residues and disease relevance (Fig. 4). The system attempts direct manuscript URL when available, DOI resolution via doi.org, PMC open access versions, and PubMed abstract pages as final fallback.

The RESTful API implements 19 endpoints following OpenAPI 3.0 specification. Search and listing endpoints implement pagination via page and per_page query parameters, field selection via fields parameter enabling sparse retrieval of required columns without transferring complete records, and response format selection via format query parameter supporting JSON (default) and CSV export.

Bulk export functionality provides complete database snapshots in two standardized formats. CSV export uses UTF-8 encoding with header row defining field names, quoted field values preventing delimiter ambiguity. JSON export implements nested structure with array of objects preserving hierarchical relationships and metadata, conforming to JSON Schema validation specification. All exports include SHA-256 checksums for data integrity verification, version identifiers linking export to database release (semantic versioning), and timestamp documentation for reproducibility. Error responses follow standard FastAPI HTTPException format providing HTTP status codes, error types, and descriptive messages for debugging.

The API implements ETags for conditional requests, enabling clients to cache responses and request updates only when data modified. Response compression via gzip reduces payload sizes across typical API calls. Error responses follow RFC 7807 Problem Details specification providing machine-readable error information with type URIs, human-readable titles, detailed messages, and suggested corrective actions.

### Comparative analysis and database coverage

**Figure 5:**
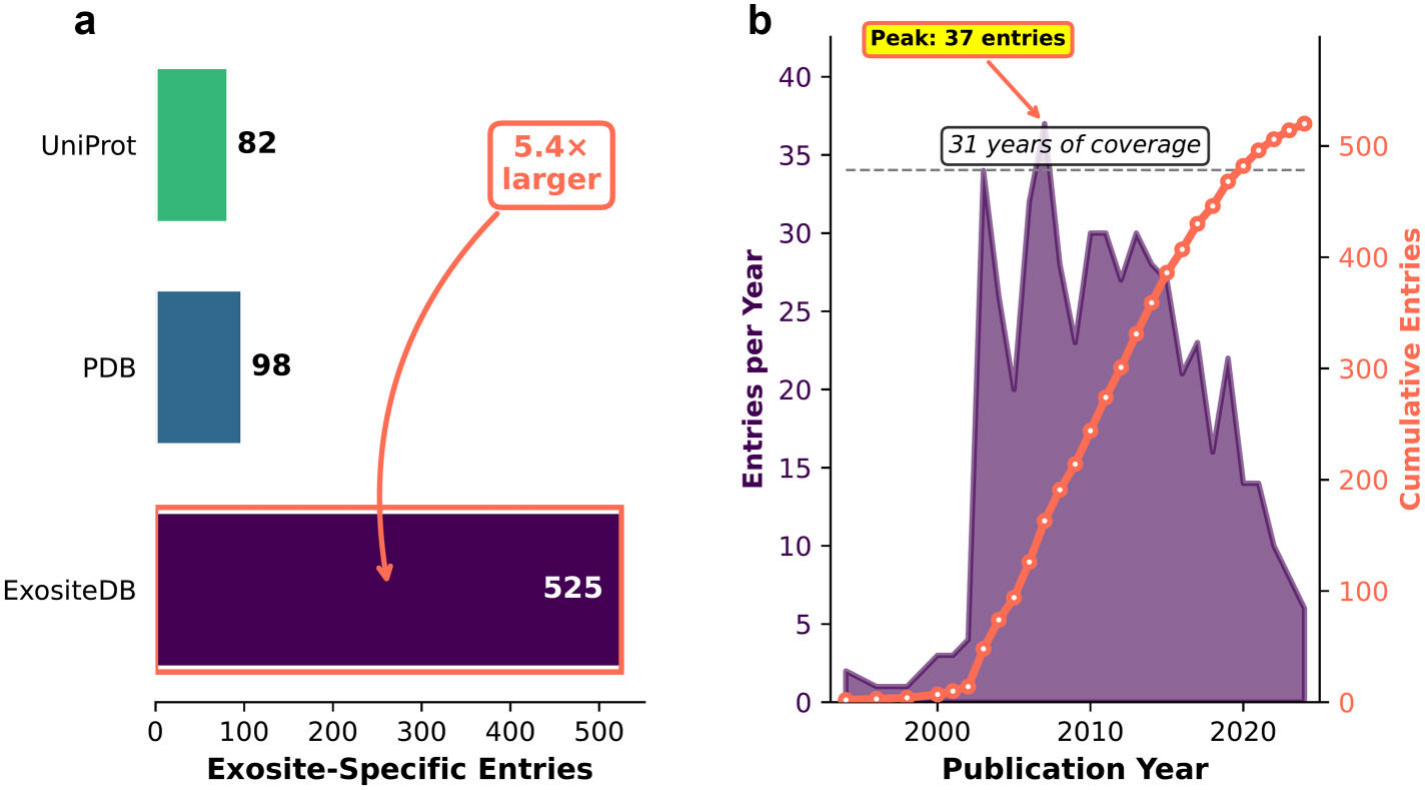
Database Coverage and Temporal Analysis. (a) Exosite-specific entry counts across databases: ExositeDB (525 entries), PDB full-text search (98 entries), and UniProt keyword search (82 entries), demonstrating 5.4$\times$ larger coverage compared to existing resources. (b) Temporal distribution showing annual entry counts (bars) and cumulative growth (overlaid cumulative line) spanning 31 years (1994–2024), with peak productivity of 37 entries in 2007.

ExositeDB offers broader coverage compared to existing resources. Comparative analysis against generalist protein databases (PDB [30,42] and UniProt [32,41]) via keyword searches for ‘exosite’ reveals 5.4-fold greater coverage relative to PDB (525 vs. 98 entries) and 6.4-fold greater coverage relative to UniProt (525 vs. 82 entries), with complete provenance tracking and systematic quality assessment (Fig. 5).

While several databases curate related biological phenomena, ExositeDB addresses a distinct niche. The Allosteric Database and AlloSite focus on allosteric regulation through ligand-induced conformational changes [34], capturing regulatory sites where binding modulates catalytic activity. ExositeDB complements these resources by specifically curating secondary binding sites that primarily mediate macromolecular partner recruitment rather than allosteric modulation, though functional overlap exists for sites exhibiting both properties. Binding databases such as PDBbind [50] and BioLIP [51] provide extensive ligand–protein interaction data but do not systematically distinguish exosites from orthosteric sites or classify binding sites by functional role [30, 31]. To our knowledge, no existing database provides dedicated exosite-specific curation with residue-level annotations, systematic confidence scoring, and explicit functional classification distinguishing partner recruitment sites from allosteric regulatory sites. ExositeDB therefore fills a critical gap in the database ecosystem, enabling focused analysis of protein recognition surfaces that mediate specificity through partner accommodation rather than catalytic modulation.

Literature coverage analysis shows broad coverage across journal categories and impact factors. High-impact journals (IF *>* 10) contribute 30% of entries, while specialized biochemistry and structural biology journals provide domain-specific depth (Supplementary Table S3). Temporal analysis reveals peak publication activity in 2007 (37 entries annually), preceded by a notable increase beginning in 2003. This peak likely reflects converging factors in the mid-2000s: the maturation of high-throughput structural biology techniques, growing pharmaceutical interest in allosteric drug discovery as an alternative to orthosteric site targeting, and intensive characterization of model systems, such as thrombin exosites, that established methodological frameworks subsequently applied to other proteins. Research activity has remained sustained since 2008, with 31% of entries derived from papers published since 2015.

Detailed analysis of the temporal trends reveals that the 2007 peak (37 entries) represents the culmination of systematic exosite characterization across the blood coagulation cascade, with 52% of entries from the peak period (2005–2009) representing trypsin-like serine proteases. Thrombin alone contributed 26 entries during this period, followed by coagulation factors X, IX, and XIII. This concentration reflects the intensive study of haemostasis mechanisms enabled by advances in structural biology (routine sub-2.0 Å co-crystal structures) and binding kinetics (surface plasmon resonance, fluorescence anisotropy).

## Usage examples

To demonstrate the practical utility of ExositeDB, we present several usage scenarios spanning computational drug discovery, machine learning, and structural biology applications.

### Programmatic access via REST API

The following examples illustrate common API usage patterns:

**Example 1: Retrieve all high-confidence thrombin exosites**

GET /api/search?protein_name=thrombin& confidence_tier=HIGH

Returns 23 entries with confidence scores $\ge$0.80, including complete residue annotations, PDB references, and literature citations.

**Example 2: Export exosites for a protein family**

GET /api/entries?family=serine_protease& format=csv

Returns 263 entries spanning trypsin-like serine proteases in CSV format suitable for spreadsheet analysis.

**Example 3: Fetch structural data for machine learning**

GET /api/entries?has_pdb=true&fields=residues, pdb_id,confidence

Returns sparse records containing only residue positions, PDB identifiers, and confidence scores for 395 structurally-characterized entries.

### Machine learning applications

ExositeDB provides curated training data for computational exosite prediction. The 395 entries with PDB structural coverage enable:

- **Residue-level classification:** Binary classification of surface residues as exosite-forming vs. non-exosite using sequence and structural features.
- **Transfer learning:** Fine-tuning protein language models on exosite-specific tasks using validated residue annotations as supervised labels.
- **Structure-based prediction:** Training graph neural networks on protein contact graphs with exosite residue labels.

The confidence scoring enables stratified training strategies, using High confidence entries for initial training and Medium confidence entries for validation.

### Structure-based drug discovery

ExositeDB supports rational drug design workflows:

- **Target identification:** Search for proteins with characterized exosites in therapeutic areas of interest.
- **Binding site mapping:** Extract residue-level annotations for structure-based virtual screening and molecular docking.
- **Selectivity analysis:** Compare exosite residue conservation across protein family members to identify selective targeting opportunities.

## Discussion

### A scalable framework for biological curation

ExositeDB shows that AI-assisted methodologies can achieve thorough literature coverage while maintaining scientific accuracy. Our three-pass gpt-4o framework enables scalable knowledge extraction, with multi-tier confidence scoring and expert validation ensuring quality control at each stage.

The four-component confidence scoring system provides systematic, reproducible quality assessment. Unlike binary validation approaches, the weighted multidimensional framework produces transparent, quantitative metrics reflecting the complexity of biological evidence. The hierarchical weighting scheme prioritizes experimental and structural evidence while incorporating literature consistency and terminology precision.

### Conceptual distinction: exosites vs. allosteric sites

The functional classification of secondary binding sites as ‘exosites’ vs. ‘allosteric sites’ warrants clarification, as these categories represent conceptually distinct yet overlapping phenomena. Exosites are defined by their primary biological role in recruiting, positioning, and accommodating macromolecular partners, substrates, or cofactors required for protein function. The fibrinogen-binding exosite (exosite I) on thrombin exemplifies this classification: fibrinogen recognition and positioning at this surface is essential for substrate specificity and catalytic processing, with the exosite serving as a macromolecular recruitment platform [9,10]. Conversely, allosteric sites modulate catalytic or functional activity through conformational transmission, with binding events at distant locations influencing active site geometry or dynamics [7,8]. Regulation rather than recognition constitutes their defining characteristic.

Functional overlap occurs when exosite occupancy triggers conformational changes that modify catalytic efficiency or substrate selectivity, integrating partner recruitment with allosteric regulation. The heparin-binding exosite (exosite II) on thrombin demonstrates this duality: heparin binding recruits antithrombin to the protein surface (exosite function) while simultaneously enhancing the rate of thrombin inhibition through conformational propagation (allosteric function) [9]. ExositeDB classification prioritizes the dominant biological role, assigning ‘exosite’ designation when partner recruitment and positioning represent the primary phenotype, while acknowledging that secondary allosteric effects may occur. This functional taxonomy enables systematic curation while recognizing the complexity of protein regulatory surfaces. Sites exhibiting equivalent contributions from both mechanisms are annotated with dual classification, preserving biological nuance while maintaining curated categories suitable for computational analysis. The companion review [13] provides comprehensive discussion of structural determinants distinguishing these regulatory paradigms and their implications for therapeutic targeting strategies.

### Quality assessment and biological validation

The mean score of 0.634 reflects incomplete multi-methodological validation for many entries, variable structural data quality despite substantial PDB coverage, and heterogeneous reporting standards reducing literature consistency scores. Approximately 41% of entries derive from pre-2010 literature when multi-method validation was less common.

Component independence analysis confirms the complementary nature of individual scoring dimensions, with experimental methodology and structural validation showing moderate correlation (*r* = 0.37) that indicates related but distinct quality assessments. The temporal improvement in confidence scores over time (from 0.65 $\pm$ 0.18 in 2000–2010 to 0.72 $\pm$ 0.16 in 2020–2024) shows the scoring system’s sensitivity to advancing experimental methodologies while preserving historical data value for thorough literature coverage.

### Integration with computational prediction

The confidence-stratified dataset curated in ExositeDB provides training data for computational prediction of exosites. In the companion study by Omage et al. [36], STINGExoFind shows that machine learning frameworks utilizing three-dimensional structural descriptors from the STING suite (SDL: Most Relevant Nanoenvironment Descriptors) achieve exosite prediction across diverse protein families. These geometry-dependent local environment descriptors capture solvent accessibility, contact density, and curvature properties at the residue level, distinguishing exosite-forming residues from other surface positions. This approach builds on our established methodology for allosteric site prediction [48,49], where SDL descriptors demonstrated strong discriminative power for identifying functional surface sites through binary residue classification.

The confidence-stratified architecture of ExositeDB enables structured machine learning workflows: High-confidence entries ($\ge$0.80, *n* = 87) serve as gold-standard positive examples for model training, Medium-confidence entries (0.60–0.79, *n* = 202) provide validation sets for hyperparameter optimization, while Low-confidence entries (*<*0.60, *n* = 236) can be leveraged for semi-supervised learning or uncertainty quantification. The published STINGExoFind framework [36] reveals that prediction performance varies across protein families, with well-represented families achieving higher accuracy due to greater training data availability, underscoring the importance of expanding database coverage across underrepresented protein classes. Future extensions integrating protein language model embeddings (e.g. ESM-2) with structural descriptors may further enhance prediction capabilities by combining evolutionary conservation signals with geometric features. Because exosite geometry and composition differ across protein families, family-specific models trained on ExositeDB subsets are expected to outperform single global classifiers, reinforcing the need for broad, balanced coverage as the database grows.

### ExositeDB-guided drug discovery: evidence tiers and practical applications

The multi-tier confidence scoring system in ExositeDB enables strategic utilization of entries based on research objectives and risk tolerance. High-confidence exosites represent validated targets suitable for immediate structure-based drug design, virtual screening campaigns, or antibody epitope selection without requiring additional experimental confirmation. Medium-confidence entries provide credible hypotheses for exploratory studies, hit validation, or lead optimization when structural data exists but multi-method validation remains incomplete. Low-confidence entries serve as literature-supported candidates requiring orthogonal experimental validation before committing substantial resources to therapeutic development.

Thrombin exemplifies the practical utility of detailed exosite annotation for therapeutic targeting. ExositeDB contains 178 thrombin entries documenting two well-characterized exosites with distinct functions and druggability profiles. Exosite I (anion-binding exosite, fibrinogen recognition) has been successfully targeted by multiple therapeutic modalities: bivalent direct thrombin inhibitors (bivalirudin, hirudin, desirudin, and lepirudin) that simultaneously engage the active site and exosite I, achieving regulatory approval for anticoagulation in specific clinical contexts [18–21]. The NU172 aptamer represents exosite-selective intervention, reported to selectively target exosite II without active site engagement, demonstrating rapid onset/offset kinetics suitable for acute interventions and reversibility via complementary antidote oligonucleotides [16,17]. PAR1 (protease-activated receptor 1) peptide mimetics targeting thrombin exosite I illustrate how understanding exosite-substrate interactions informs peptidomimetic design for pathway-selective modulation.

The therapeutic advantages of exosite targeting emerge clearly from these examples: selectivity arising from lower evolutionary conservation relative to catalytic sites (thrombin exosite I shows reduced conservation compared to active site residues among related trypsin-like proteases), preserved physiological regulation (exosite inhibitors do not block basal catalytic activity against all substrates), reduced resistance evolution (no selective pressure on catalytic machinery), and modality flexibility (small molecules for pocket-containing exosites, biologics for protein-interaction surfaces). Matrix metalloproteinase (MMP) exosites demonstrate similar principles: MMP-12 macrophage elastase possesses an S1’ subsite exosite mediating substrate selectivity [24], enabling design of MMP-12-selective inhibitors that avoid off-target effects on other MMPs implicated in tissue homeostasis. ExositeDB residue-level annotations for 45 MMP exosite entries provide the structural foundation for such selectivity-focused inhibitor design.

The database further supports fragment-based drug discovery and hit-to-lead optimization through detailed binding site annotations. High-confidence entries include experimentally validated contact residues, enabling focused molecular docking screens and structure–activity relationship interpretation. For proteins lacking direct drug precedent, homology-based strategies leveraging well-characterized exosites in related family members provide starting hypotheses. The integration of confidence scores with structural annotations allows researchers to prioritize targets balancing biological validation (confidence) with structural tractability (PDB coverage, pocket geometry) for cost-effective therapeutic discovery campaigns.

### Limitations and future directions

Several limitations inform ongoing development priorities. The current release contains limited coverage of literature published after 2022, reflecting the time-intensive nature of expert validation during initial database construction. This temporal gap will be addressed through continuous literature surveillance pipelines currently under development, incorporating automated weekly PubMed queries with AI-assisted triage and expert review workflows. Post-2022 publications describing novel exosites or expanding characterization of existing entries will be prioritized for expedited curation, with quarterly database releases ensuring timely incorporation of recent findings.

Dataset composition exhibits substantial bias towards well-characterized protein families, particularly blood coagulation proteases (50% of entries). This distribution reflects historical research priorities rather than biological prevalence, with proteases benefiting from extensive pharmaceutical investment, methodological accessibility (soluble proteins amenable to crystallography), and clinical validation of exosite-targeting drugs. Mitigation strategies include: (i) systematic expansion to underrepresented families through targeted literature searches in kinases, phosphatases, membrane proteins, and metabolic enzymes; (ii) integration of AlphaFold-predicted structures (*>*200 million proteins) to enable exosite annotation for proteins lacking experimental structures, with appropriate confidence score adjustments reflecting computational model uncertainty; and (iii) community-driven curation mechanisms described below. The published STINGExoFind prediction framework [36] provides an avenue for genome-scale exosite identification that can guide experimental validation priorities towards underrepresented protein classes.

The absence of validated negative examples (confirmed non-exosite binding sites) represents a current limitation for supervised machine learning applications requiring both positive and negative training instances. While ExositeDB provides high-quality positive examples, computational prediction methods would benefit from curated datasets distinguishing exosites from orthosteric binding sites and non-functional surface patches. Future development will incorporate systematic annotation of negative controls through three approaches: (i) orthosteric sites explicitly annotated as ‘active site’ rather than exosite in source literature; (ii) protein surfaces with no documented binding function based on extensive mutagenesis studies; and (iii) computationally predicted non-binding surfaces validated through molecular dynamics simulations and experimental testing. This negative dataset will enable more robust classifier training and reduce false-positive rates in genome-scale exosite prediction.

Community engagement through collaborative curation mechanisms will address coverage limitations and accelerate database growth. Planned implementation includes: (i) GitHub-based submission platform enabling researchers to contribute novel exosite characterizations with supporting evidence, following templates that ensure data quality and completeness; (ii) expert review workflow where community contributions undergo validation by domain specialists before database integration, maintaining scientific rigour while leveraging distributed expertise; (iii) version-controlled data releases with detailed change logs documenting community contributions and their validation status; and (iv) contributor recognition through explicit attribution in database records and annual acknowledgment of high-impact submissions. This model, successfully employed by UniProt and other community databases, enables scalable growth while preserving curation standards. Initial community pilot will focus on expanding kinase and membrane protein coverage through targeted outreach to relevant research communities.

Integration of emerging structural biology methodologies represents ongoing development priorities. AlphaFold2 and AlphaFold3 structure predictions provide structural coverage for proteins lacking experimental structures, enabling exosite annotation guided by predicted conformations. Confidence score frameworks will incorporate model quality metrics (pLDDT, PAE) to appropriately weight computationally predicted structures relative to experimental data. Cryo-EM structures of membrane protein complexes provide opportunities to expand ExositeDB beyond the current bias towards soluble, crystallography-amenable proteins. Integrative structural biology approaches combining experimental and computational data will be systematically incorporated as these methodologies mature and validation frameworks emerge.

Functional validation of exosites through high-throughput mutagenesis, deep mutational scanning, and yeast display experiments generates residue-level functional maps that complement literature curation. Systematic integration of these datasets, when publicly available, will enhance confidence scoring and provide orthogonal validation of literature-derived annotations. Collaboration with structural genomics initiatives and pharmaceutical screening campaigns provides pathways for incorporating unpublished functional data while respecting proprietary constraints through tiered data release mechanisms.

## Conclusions

ExositeDB is, to our knowledge, the first curated database dedicated to protein exosites. It contains 525 expert-validated records spanning 280 proteins and 395 PDB structures, each annotated at residue-level resolution with full literature provenance and four-component confidence scoring. By filling the gap between generalist structural repositories and the growing body of exosite literature, ExositeDB supplies the curated data layer needed for both mechanistic analysis and computational prediction. Together with the STINGExoFind prediction tool [36] and the companion structural review [13], the database forms an integrated triad covering data, prediction, and biological interpretation for exosite-driven drug discovery. As the database expands through community curation and broader protein family coverage, we expect ExositeDB to serve as a reference resource for the rational design of selective exosite-targeting therapeutics.

## Supplementary Material

baag031_Supplemental_Files

## Data Availability

Database Access: ExositeDB is freely available at https://www.exosite.cbi.cnptia.embrapa.br/ with interactive web interface, bulk data downloads in CSV/JSON formats, and comprehensive REST API access. All database content is released under Creative Commons Attribution 4.0 International License (CC BY 4.0), permitting reuse with attribution. API Documentation: Interactive API documentation following OpenAPI 3.0 specification is available at https://www.exosite.cbi.cnptia.embrapa.br/docs with executable examples and downloadable schema enabling client code generation using standard OpenAPI tools.
