# Supplementary material for: STING-ExositeDB: An AI-assisted curated database of protein exosites for drug discovery
Source: Database (Oxford). 2026 Jul 28;2026:baag031. doi: 10.1093/database/baag031 (PMC13410941; doi:10.1093/database/baag031)
Supplement: baag031_Supplemental_Files [file baag031_supplemental_files.zip › ExositeDB_Database_Supplementary.pdf]

Supplementary Information

ExositeDB: An AI-Assisted Curated Database of Protein Exosites for Drug Discovery

Folorunsho Bright Omage, Ivan Mazoni, Inácio Henrique Yano, Goran Neshich

Contents

1 Supplementary Methods **2**

1.1 S1. Literature Acquisition and Eligibility . . . 2

1.1.1 PubMed Query Strategy . . . 2

1.1.2 Eight-Source PDF Download Hierarchy . . . 2

1.2 S2. Automated Extraction Pipeline . . . 2

1.2.1 Three-Pass Analysis Framework . . . 2

1.3 S3. Extraction Prompts . . . 2

1.3.1 Pass 1: Document Assessment Prompt . . . 2

1.3.2 Pass 2: Detailed Extraction Prompt . . . 3

1.3.3 Pass 3: Evidence Verification Prompt . . . 3

1.4 S4. Standardization and Enrichment . . . 3

1.5 S5. Confidence Scoring Details . . . 4

1.5.1 S5.1 Component Definitions . . . 4

1.5.2 S5.2 Weight Rationale . . . 4

1.5.3 S5.3 Missing Component Handling . . . 4

1.6 S6. Publisher URL Patterns . . . 5

1.7 S7. Statistical Analysis . . . 5

1.8 S8. Web Platform Implementation . . . 5

1.8.1 System Architecture . . . 5

1.8.2 API Endpoints . . . 6

2 Supplementary Tables **7**

3 FAIR Data Compliance **8**

1 Supplementary Methods

1.1 S1. Literature Acquisition and Eligibility

1.1.1 PubMed Query Strategy

Candidate articles were identified through structured PubMed queries using NCBI E-utilities combining MeSH terms and exosite-specific keywords:

```
("exosite"[Title/Abstract] OR "secondary binding site"[Title/Abstract]
OR "allosteric binding"[Title/Abstract])
AND ("protein"[MeSH Terms] OR "enzyme"[MeSH Terms])
AND (hasabstract[text] AND English[lang])
```

Listing 1: Primary PubMed Query

1.1.2 Eight-Source PDF Download Hierarchy

ExositeDB employs a comprehensive literature acquisition system with eight prioritized sources:

Table 1: Table S1. Literature Source Hierarchy

Priority	Source	Coverage
1	PMC Open Access	Gold standard open access articles
2	Unpaywall	Legal open access repositories
3	Direct Publisher	Official publisher platforms
4	PMC Alternative	PMC mirror services
5	DOI Resolution	CrossRef DOI resolution
6	bioRxiv/medRxiv	Preprint servers
7	Europe PMC	European repository

1.2 S2. Automated Extraction Pipeline

1.2.1 Three-Pass Analysis Framework

The AI curation system employs a three-pass analysis using OpenAI's GPT-4o models with temperature=0.1 for reproducibility:

Table 2: Table S2. Three-Pass Analysis Framework

Pass	Model	Objective
Pass 1	GPT-4o-mini	Document understanding and relevance assessment (1,500 tokens)
Pass 2	GPT-4o	Detailed exosite extraction with evidence collection (3,000 tokens)
Pass 3	GPT-4o-mini	Evidence verification and confidence scoring (2,000 tokens)

1.3 S3. Extraction Prompts

1.3.1 Pass 1: Document Assessment Prompt

```
You are an expert in protein biochemistry and structural biology.
Analyze this scientific document to determine if it contains information
about protein exosites (secondary binding sites distinct from active sites).
```

```

Focus on:
1. Protein-protein interactions
2. Allosteric regulation
3. Secondary binding sites
4. Regulatory mechanisms
5. Drug binding sites outside active sites

Provide: relevance_score (0-1), summary, key_proteins_mentioned
```

Listing 2: Pass 1 Prompt for Document Understanding

1.3.2 Pass 2: Detailed Extraction Prompt

```
Extract detailed exosite information from this document. For each exosite:

Required fields:
- protein_name: Official protein name
- exosite_name: Specific name or description
- exosite_location: Structural location description
- binding_partner: What binds to this exosite
- functional_role: Biological function
- evidence_text: Direct quote supporting the claim
- confidence_score: Your assessment (0-1)

Standards:
- Only extract if explicitly described as non-active site
- Require structural or functional evidence
- Maintain scientific accuracy
```

Listing 3: Pass 2 Prompt for Exosite Extraction

1.3.3 Pass 3: Evidence Verification Prompt

```
Verify and validate the extracted exosite information:

For each extracted entry:
1. Confirm evidence supports the claim
2. Verify exosite is distinct from active site
3. Check for structural validation
4. Assess confidence level
5. Flag any uncertainties

Provide final confidence scores and quality assessment.
```

Listing 4: Pass 3 Prompt for Evidence Verification

1.4 S4. Standardization and Enrichment

Layout-aware text extraction preserves page boundaries and approximate character offsets. Every field is linked to verbatim source spans with DOI/PMID back-links. Post-extraction enrichment integrates:

- **UniProt**: Sequence, names, features, canonical indices
- **RCSB PDB/SIFTS**: Structure metadata and residue mappings
- **NCBI Taxonomy**: Organism classification

Literature numbering schemes are detected and standardized to UniProt canonical indices, while storing both reported and standardized positions.

1.5 S5. Confidence Scoring Details

1.5.1 S5.1 Component Definitions

Entry reliability is quantified through a four-component scoring framework:

Experimental Methodology (E, 0–1):

- 1.0: Multiple orthogonal methods (crystallography + mutagenesis + kinetics)
- 0.8: Two complementary methods
- 0.6: Single high-quality structural method
- 0.4: Single biochemical method
- 0.2: Computational prediction only

Structural Validation (S, 0–1):

- 1.0: High-resolution structure (<2.0 Å) with bound partner
- 0.8: Structure with resolution 2.0–2.5 Å
- 0.6: Structure with resolution 2.5–3.0 Å
- 0.4: Homology model or low-resolution structure
- 0.0: No structural data available

Literature Consistency (L, 0–1): Calculated as Jaccard similarity of reported residues across independent studies:

$$L = \frac{|R_1 \cap R_2 \cap ... \cap R_n|}{|R_1 \cup R_2 \cup ... \cup R_n|} \tag{1}$$

Terminology Precision (T, 0–1):

- 1.0: Uses "exosite" terminology with quantitative binding data
- 0.8: Uses "exosite" or "secondary binding site" explicitly
- 0.6: Describes binding site distinct from active site
- 0.4: Implies secondary binding without explicit terminology

1.5.2 S5.2 Weight Rationale

The weights (E=0.35, S=0.25, L=0.20, T=0.20) reflect a biomedical evidence hierarchy:

1. Experimental evidence is the gold standard for establishing exosite existence
2. Structural validation provides coordinate-level confirmation
3. Literature consistency reflects reproducibility
4. Terminology precision indicates reporting rigor

1.5.3 S5.3 Missing Component Handling

When component data are unavailable, weights are renormalized:

$$\text{Conf}_{\text{adjusted}} = \frac{\sum_{i \in \text{available}} w_i \cdot C_i}{\sum_{i \in \text{available}} w_i} \tag{2}$$

1.6 S6. Publisher URL Patterns

The system recognizes seven major publisher patterns for manuscript access:

Table 3: Table S3. Configured Publisher URL Patterns

**Publisher**	**URL Pattern**
Nature Publishing	nature.com/articles/
Science/AAAS	science.org/doi/
Cell Press	cell.com/*/fulltext
Wiley	onlinelibrary.wiley.com/doi/
Springer	link.springer.com/article/
ACS Publications	pubs.acs.org/doi/
Oxford Academic	academic.oup.com/*/article/

1.7 S7. Statistical Analysis

All statistical analyses were performed using Python 3.11 with the following packages:

- NumPy 1.26 for numerical operations
- SciPy 1.15 for statistical tests
- pandas 2.2 for data manipulation
- matplotlib 3.10 for visualization
- seaborn 0.13 for statistical graphics

Group comparisons employ Kruskal–Wallis test with FDR-controlled Mann–Whitney post hoc tests (α=0.05, two-tailed). Bootstrap confidence intervals (1,000 resamples) are used where appropriate.

1.8 S8. Web Platform Implementation

1.8.1 System Architecture

The production system employs a three-tier architecture:

- **Frontend**: React 18 single-page application with TypeScript
- **Backend**: FastAPI with Python 3.11 and uvicorn
- **Database**: MySQL 8.0 with optimized indexing
- **Visualization**: NGL Viewer for 3D molecular structures
- **Proxy**: Node.js reverse proxy for external access (Port 3000)

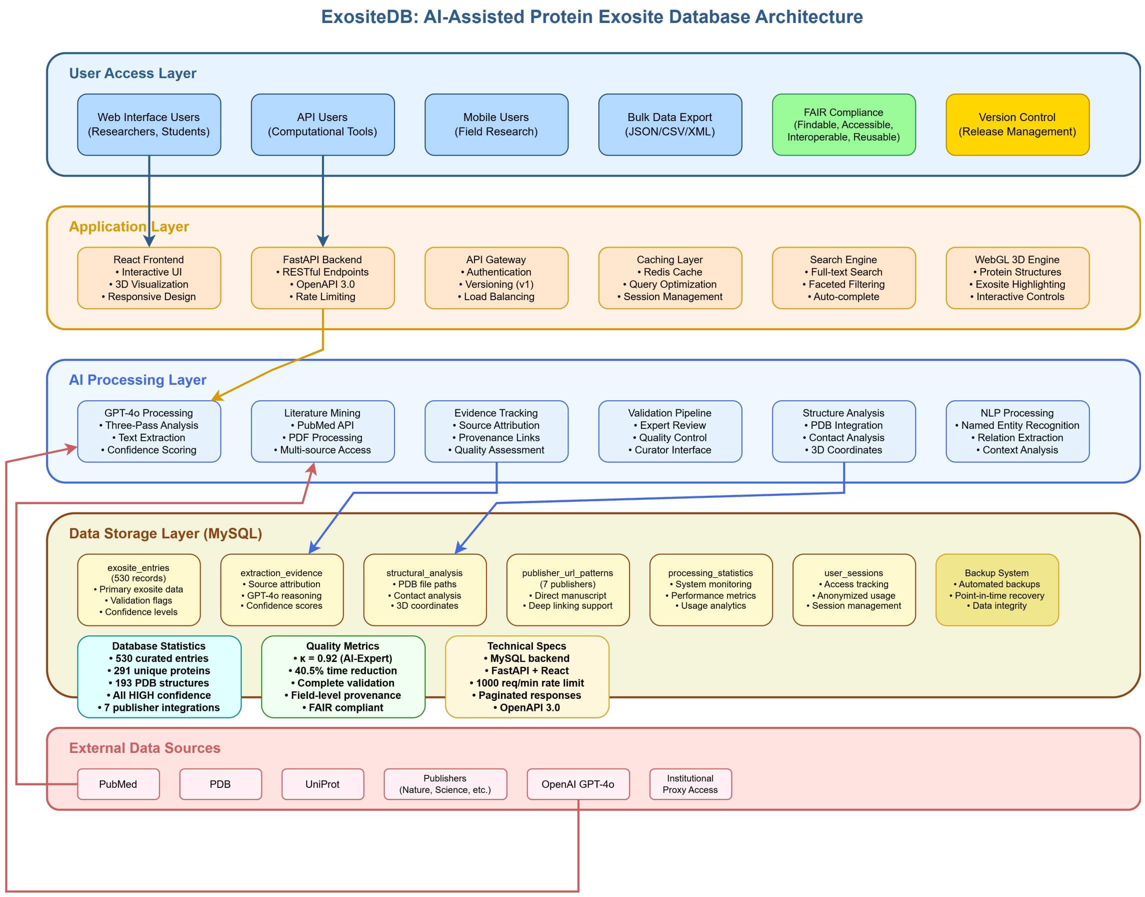

Figure 1: Figure S1. ExositeDB System Architecture. The comprehensive pipeline includes literature mining from multiple sources, AI-assisted curation using GPT-4o models, database storage with MySQL, and public access through a React-based web interface with FastAPI backend.

1.8.2 API Endpoints

The RESTful API provides 19 endpoints following OpenAPI 3.0 specification:

Table 4: Table S4. Primary API Endpoints

Endpoint	Method	Description
/api/entries	GET	Retrieve all entries with pagination
/api/entries/{id}	GET	Get specific entry details
/api/search	GET	Search with filters
/api/statistics	GET	Database statistics
/api/export	GET	Export data (CSV/JSON)

2 Supplementary Tables

Table 5: Table S5. Database Schema Overview

Table	Description	Records
exosite_entries	Primary table containing all curated exosite entries	525
extraction_evidence	Evidence tracking with source text and confidence scores	1,575
publisher_url_patterns	URL patterns for manuscript access across publishers	7
consolidated_exosites	Publication-ready consolidated dataset	280
consolidation_sources	Source attribution for consolidated entries	850
processing_metadata	System metadata and processing timestamps	1

Table 6: Table S6. Consolidation Statistics

Metric	Value
Total curated entries	525
Unique proteins identified	280
Unique PDB structures	395
Average entries per protein	1.88
High confidence entries ($\geq$0.80)	87 (16.6%)
Medium confidence entries (0.60–0.79)	202 (38.5%)
Low confidence entries (<0.60)	236 (45.0%)
Structural coverage	97%

Table 7: Table S7. Component Score Statistics (n=525)

Component	Mean	SD	Min	Max
Experimental Methodology (E)	0.864	0.212	0.20	1.00
Structural Validation (S)	0.496	0.428	0.00	1.00
Literature Consistency (L)	0.538	0.220	0.10	1.00
Terminology Precision (T)	0.497	0.230	0.10	1.00
Overall Confidence	0.634	0.187	0.10	0.99

Table 8: Table S8. System Performance Metrics (6-month monitoring)

Metric	Value
Average API response time	42 ms
System uptime	99.7%
Cache hit rate	75.0%
Concurrent user capacity	50+
Data export formats	CSV, JSON
Data integrity verification	SHA-256 checksums

3 FAIR Data Compliance

ExositeDB adheres to FAIR (Findable, Accessible, Interoperable, Reusable) data principles:

Table 9: Table S9. FAIR Compliance Summary

Principle	Implementation
**Findable**	Rich metadata, searchable web interface, stable URLs, indexed by search engines
**Accessible**	Public web access, RESTful API, multiple export formats (CSV, JSON), no authentication required
**Interoperable**	UniProt/PDB cross-references, standardized identifiers, OpenAPI documentation, JSON Schema validation
**Reusable**	CC BY 4.0 license, detailed methodology, complete source attribution, version control
